# *Artemisia iwayomogi* Extract Attenuates High-Fat Diet-Induced Obesity by Decreasing the Expression of Genes Associated with Adipogenesis in Mice

**DOI:** 10.1155/2013/915953

**Published:** 2013-01-17

**Authors:** Yeji Choi, Yasuko Yanagawa, Sungun Kim, Wan Kyunn Whang, Taesun Park

**Affiliations:** ^1^Department of Food and Nutrition, Yonsei University, 50 Yonsei-ro, Seodaemun-gu, Seoul 120-749, Republic of Korea; ^2^College of Pharmacy, Chung-Ang University, 221 Heuksuk-Dong, dongjak-gu, Seoul 156-756, Republic of Korea

## Abstract

The objective of the present study was to determine whether *Artemisia iwayomogi* (AI) extract reduces visceral fat accumulation and obesity-related biomarkers in mice fed a high-fat diet (HFD), and if so, whether these effects are exerted by modulation of the expression of genes associated with adipogenesis and inflammation. AI extract supplementation for 11 weeks significantly prevented HFD-induced increments in body weight, visceral adiposity, adipocyte hypertrophy, and plasma levels of lipids and leptin. Additionally, AI extract supplementation resulted in downregulation of adipogenic transcription factors (PPAR**γ**2 and C/EBP**α**) and their target genes (CD36, aP2, and FAS) in epididymal adipose tissue compared to the HFD alone. The AI extract effectively reversed the HFD-induced elevations in plasma glucose and insulin levels and the homeostasis model assessment of insulin resistance index. Furthermore, the extract significantly decreased gene expression of proinflammatory cytokines (TNF**α**, MCP1, IL-6, IFN**α**, and INF**β**) in epididymal adipose tissue and reduced plasma levels of TNF**α** and MCP1 as compared to HFD alone. In conclusion, these results suggest that AI extract may prevent HFD-induced obesity and metabolic disorders, probably by downregulating the expression of genes related to adipogenesis and inflammation in visceral adipose tissue.

## 1. Introduction

Adipogenesis is the process by which mesenchymal precursor cells differentiate into adipocytes [1]. Although its presence is necessary in many ways, excess adipose tissue is associated with serious health problems such as obesity, cardiovascular disease, and type 2 diabetes [2, 3]. Presently, CCAAT/enhancer binding protein *α* (C/EBP*α*) and peroxisome proliferator-activated receptor *γ*2 (PPAR*γ*2) are considered the 2 primary transcription factors that mediate adipogenesis. It has been reported that inactivation of PPAR*γ*2 and C/EBP*α* in adipose tissue protects against obesity in rodent models [4–6]. Thus, potential therapeutic agents that have the ability to inhibit adipogenesis could have a profound impact as a strategy for preventing obesity and related metabolic disorders.

Adipose tissue not only serves as an organ for energy storage, but also as an endocrine organ by releasing various inflammatory cytokines such as tumor necrosis factor *α* (TNF*α*) and interleukin- (IL-)6 [7–9]. Proinflammatory molecules produced by adipose tissue have been implicated as active participants in the development of inflammation and the increased risk of obesity-related insulin resistance [9–11]. Increased production of monocyte chemoattractant protein 1 (MCP1), interferon (IFN) *α*, IFN*β*, TNF*α*, and IL-6 in adipose tissue has been reported in animal models of obesity [8–10, 12]. Therefore, therapeutic agents that attenuate proinflammatory cytokines may prove useful in the medical management of obesity-induced inflammation.

*Artemisia iwayomogi* (AI), a member of *Compositae*, is a perennial herb easily found throughout Korea. It has been used as a traditional medicine and is known to have antiallergic, antiapoptotic, and antioxidant effects [13–16]. It has been reported that a carbohydrate fraction from AI suppresses spontaneous or 2,3,7,8-tetrachlorodibenzo-p-dioxin-induced apoptotic death of mouse thymocytes [14, 15]. *Artemisia iwayomogi* extract displays scavenging activity of peroxynitrite, a potent cytotoxic oxidant formed by the reaction between nitric oxide and superoxide radicals [16]. In addition, to our knowledge, few studies have described the beneficial effects of AI in high-fat diet-(HFD-) fed obese rodents. One study reported that oral administration of AI extract significantly reduced serum lipid levels in HFD-fed rats [17]. A recent study by Cho et al. reported that oral administration of AI extract to HFD-fed mice provoked upregulation of PPAR*β* and its target genes involved in fatty acid oxidation in the skeletal muscle [18]. To date, however, no study has assessed the protective effects of AI extract on adipose tissue dysfunction in diet-induced obese animal models. Therefore, the present study aimed to investigate whether AI extract could reduce visceral fat accumulation and improve obesity-related biomarkers in HFD-fed mice, and if so, whether these effects were exerted by modulation of the expression of genes associated with adipogenesis and inflammation. 

## 2. Material and Methods

### 2.1. Preparation of AI Extract

*Artemisia iwayomogi* was collected from Korean standard products in March 2008, and was identified by Professor Wan Kyun Whang of the Pharmaceutical Botany Laboratory at Chung-Ang University in Seoul, Republic of Korea, where a voucher specimen has been deposited. Dried AI (500 g) was washed in water, oven dried at 40°C, mechanically fragmented, and then was powdered in an electric mill. The powder was extracted 5 times with ethanol (powder : solvent = 1 : 5) at room temperature. After filtration, the extraction was vacuum concentrated to yield 6.25% ethanol extract (31.3 g), which was stored at −4°C until use. 

### 2.2. HPLC Analysis

Chromatography was performed using a Water HPLC system (Water Corporation, Milford, MA, USA) with an autosampler. HPLC separation was conducted using a Kromasil C18 column (4.6 mm × 250 mm, 5-*μ*m inner diameter) at 30°C with a flow rate of 1.0 mL/min using a gradient mobile phase composed of water (A) and acetonitrile (B). The mobile phase comprised an 80 : 20 mixture of component A to B as the initial condition of the chromatographic run, and component B was increased to 80% in a linear gradient in 30 min. The sample injection volume was 10 *μ*L. The identities of the compounds in the AI extract were confirmed by  ^1^H-NMR,  ^13^C-NMR, and MS, with individual purities of not less than 95%.

### 2.3. Animals and Experimental Protocol

Male C57BL/6J mice (5 weeks old) were purchased from Orient Bio (Gyeonggi-do, Republic of Korea) and were maintained in 12 h light/dark with *ad libitum* access to food and water. After a 1-week acclimatization period, the mice were divided into 3 groups (*n* = 10 per group): normal diet (ND), HFD, and 0.5% AI extract-supplemented diet (AED). The ND was a purified diet based on the AIN-76 rodent diet composition. The HFD was identical to the ND, but to which 200 g fat/kg (170 g lard plus 30 g corn oil) and 1% cholesterol had been added. The AED was identical to the HFD but contained 0.5% (w/w) AI extract. The mice were fed the experimental diets for 11 weeks. Diet consumption was monitored daily, and body weight was monitored weekly. At the end of the feeding period, mice were anesthetized with diethyl ether after an overnight fasting for 16 h, and their blood samples were collected in EDTA-coated tubes. Plasma samples were isolated by centrifugation at 4000 ×g for 20 min and stored at −80°C for subsequent analysis. Adipose and liver tissues were collected, washed with phosphate-buffered saline, and frozen at −80°C. All animal experiments were performed in accordance with the Korean Food and Drug Administration guidelines. The Institutional Animal Care and Use Committee of the Yonsei Laboratory Animal Research Center reviewed and approved the protocols. 

### 2.4. Biochemical Analysis

Plasma concentrations of triglycerides (TGs), free fatty acids (FFAs), glucose, total cholesterol, and HDL cholesterol were measured using commercial kits (Bio-Clinical System, Gyeonggi-do, Republic of Korea). LDL + VLDL cholesterol levels were calculated by subtracting HDL cholesterol from total cholesterol. Plasma insulin levels were analyzed using a mouse insulin ELISA kit (ALPCO Diagnostics, Windham, NH, USA). The homeostasis model assessment of insulin resistance (HOMA-IR) index was calculated as fasting plasma glucose concentration (mmol/L) multiplied by fasting insulin level (pmol/L) divided by 22.5. Plasma levels for leptin, TNF*α*, and MCP1 were measured using a mouse ELISA kit (ID Labs, Cambridge, MA, USA). Hepatic lipids were extracted as described by Folch et al. [19], using a chloroform-methanol mixture (2 : 1 v/v), and the dried lipid residues were dissolved in 2 mL ethanol. Concentrations of cholesterol, triglyceride, and free fatty acids in the hepatic lipid extracts were measured using the same enzymatic kits that were used for the plasma analysis. 

### 2.5. Histological Analysis

White adipose tissues (WATs) were fixed in neutral buffered formalin, embedded in paraffin, and sectioned into 5 *μ*m sections onto slides. For histology, sections were stained with hematoxylin and eosin (H&E). The sectional areas of WAT were analyzed to quantify the size of the adipocytes. 

### 2.6. RNA Extraction and Semiquantitative RT-PCR

Total RNA was isolated from the epididymal adipose tissue of each mouse with TRIzol (Invitrogen, Carlsbad, CA, USA) according to the manufacturer's instruction. Isolated RNA was quantified using a spectrophotometer, and cDNA was synthesized using reverse transcriptase (Invitrogen). The PCR was programmed as follows: 10 min at 94°C; 30–33 cycles at 94°C for 30 s, 55°C for 30 s; 72°C for 1 min; 10 min incubation at 72°C. Four microliters of each PCR reaction were mixed with 1 *μ*L of 6-fold concentrated loading buffer and loaded onto 2% agarose gel containing ethidium bromide. The GenBank accession numbers of the relevant templates and forward (F) and reverse (R) primer sequences are shown in Table 1. The measured mRNA levels were normalized to the glyceraldehyde-3-phosphate dehydrogenase (GAPDH) mRNA levels. 

### 2.7. Statistical Analysis

The data on body weight gain, plasma biochemistries, and adipocyte diameter are presented as the mean ± SEM of 10 mice. RT-PCR results are presented as the mean ± SEM of at least 3 separate experiments. All analyses were performed using SPSS (version 12.0). Data were analyzed by 1-way ANOVA, followed by Duncan's multiple range tests. *P* values <0.05 were considered significant. 

## 3. Results

### 3.1. Chromatographic Analysis of Artemisia iwayomogi Extract

**Figure 1:**
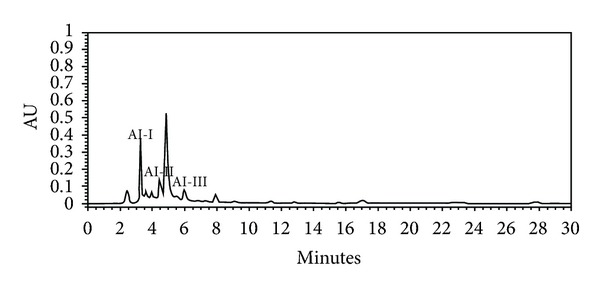
HPLC chromatogram of the *Artemisia iwayomogi* extract. The peaks were assigned based on the isolation of each compound. AI-I: scopolin; AI-II: acetophenone glycoside; AI-III: scopoletin (see Table 2).

The HPLC chromatogram revealed that scopolin (AI-I, 1.21% w/w), acetophenone glycoside (AI-II, 0.26% w/w), and scopoletin (AI-III, 0.38% w/w) were the major components among the organic molecules of the AI extract, which exhibited maximum absorbance at 280 nm (Figure 1 and Table 2). 

### 3.2. Body and Visceral Fat Pad Weights

**Figure 2:**
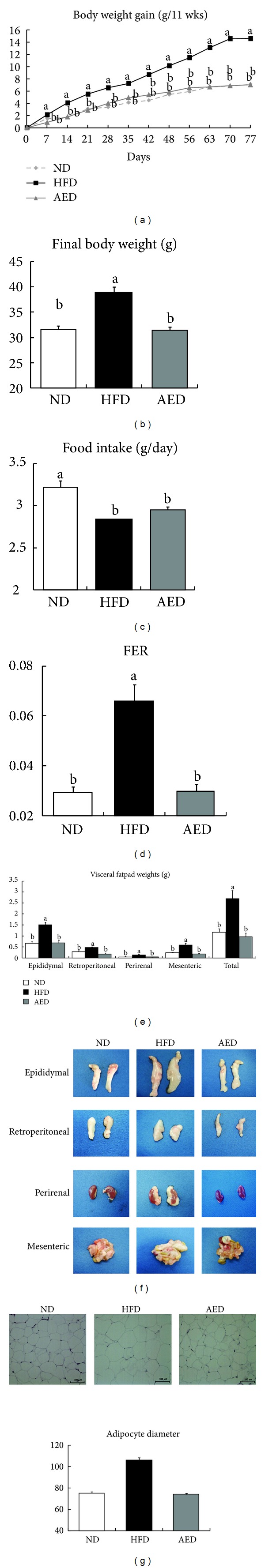
Effects of *Artemisia iwayomogi* extract supplementation on body weight gain, food efficiency ratio, and visceral fat pad weights of mice fed with high-fat diet. Mice were fed ND, HFD, or AED for 11 weeks. Changes in (a) body weight gain, (b) final body weight, (c) food intake, (d) FER, (e), (f) visceral fat pad weights, (g) representative pictures of H&E-stained fat cells from mice epididymal adipose tissue (×100), and densitometric analysis of adipocyte diameter in epididymal tissue. Data represent mean ± SEM, *n* = 10. Means without a common letter differ, *P* < 0.05. FER = (body  weight  gain  for  experimental  period  (g))/(food  intake  for  experimental  period  (g)).

AED-fed mice exhibited significantly decreased body weight gain (−52%) and final body weight (−19%) compared to HFD-fed mice without their food intake being affected (Figures 2(a)–2(c)). The food efficiency ratio (FER) was significantly lower in AED-fed mice (−54%) compared to HFD-fed mice (Figure 2(d)). The AI extract supplementation led to a significant decrease in total visceral fat pad weight (−64%) compared to the HFD alone. This was attributable to weight decreases in the epididymal (−54%), retroperitoneal (−58%), perirenal (−83%), and mesenteric (−69%) adipose depots (*P* < 0.05 for all depots; Figures 2(e) and 2(f)). The H&E sections of epididymal adipose tissues revealed that adipocyte diameter (−20%) was significantly decreased in AED-fed mice than in HFD-fed mice (Figure 2(g)). 

### 3.3. Plasma Biochemistries

**Figure 3:**
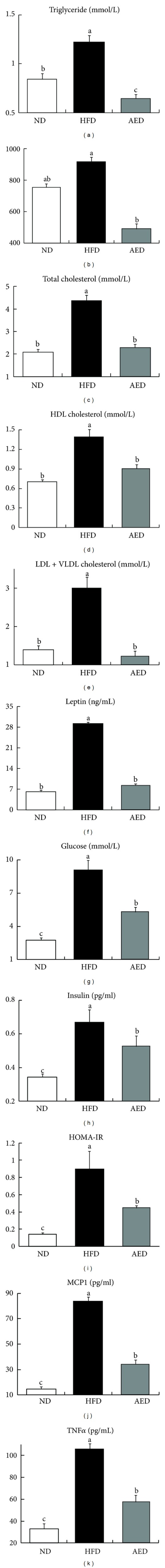
Effects of *Artemisia iwayomogi* extract supplementation on plasma levels of lipids, leptin, glucose, insulin, and proinflammatory cytokines in mice fed with high-fat diet. (a) Triglyceride, (b) free fatty acid, (c) total cholesterol, (d) HDL cholesterol, (e) LDL + VLDL cholesterol, (f) leptin, (g) glucose, (h) insulin, (i) HOMA-IR, (j) MCP1, and (k) TNF*α*. Bars represent mean ± SEM, *n* = 10. Means without a common letter differ, *P* < 0.05.

Plasma concentrations of TG (−47%), FFA (−47%), total cholesterol (−47%), HDL cholesterol (−34%), and LDL + VLDL cholesterol (−59%) were all significantly lower in AED-fed mice than in HFD-fed mice (*P* < 0.05, Figures 3(a)–3(e)). AED-fed mice had significantly lower plasma concentrations of leptin (−71%) than HFD-fed mice (Figure 3(f)). Likewise, the AI extract significantly attenuated the elevation in plasma concentrations of glucose (−42%) and insulin (−21%) in HFD-fed mice (Figures 3(g) and 3(h)). The HOMA-IR calculations revealed that the AI extract significantly decreased the HOMA-IR index (−49%) compared to HFD alone (Figure 3(i)). AED-fed mice had significantly lower plasma concentrations of MCP1 (−60%) and TNF*α* (−46%) than HFD-fed mice (Figures 3(j) and 3(k)). 

### 3.4. Hepatic Lipid Accumulation

**Figure 4:**
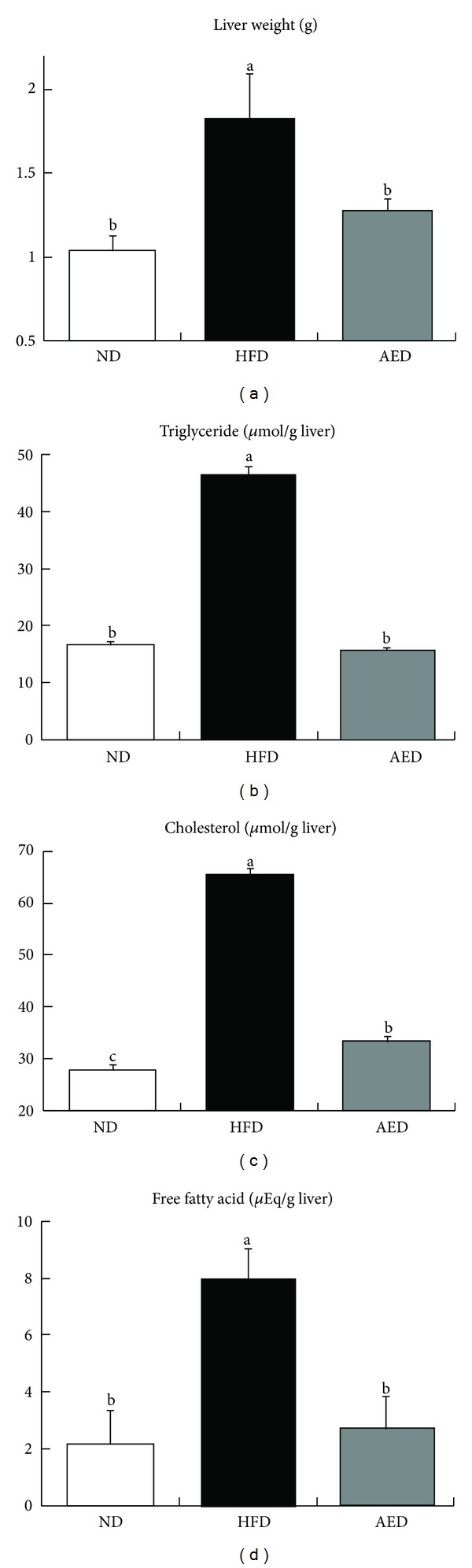
Effects of *Artemisia iwayomogi* extract supplementation on liver weights and hepatic lipid levels in mice fed with high-fat diet. (a) Liver weights and concentrations of hepatic (b) triglyceride, (c) cholesterol, and (d) free fatty acids. Means without a common letter differ, *P* < 0.05.

AED-fed mice had significantly lower liver weight (−30%) than in HFD-fed mice (Figure 4(a)). Hepatic triglyceride (−66%), cholesterol (−51%), and free fatty acid (−75%) concentrations were markedly lower in AED-fed mice than in HFD-fed mice (Figures 4(b)–4(d)). 

### 3.5. Expression of Genes Related to Adipogenesis

**Figure 5:**
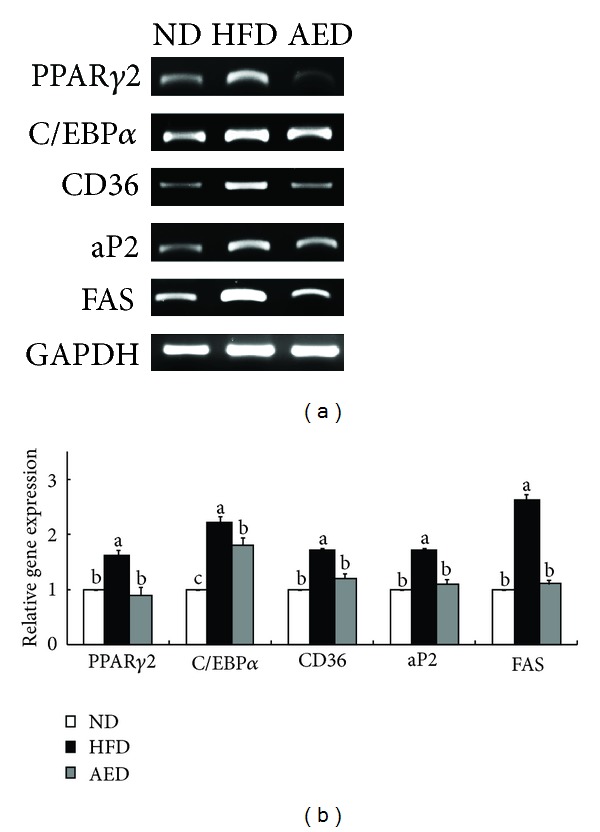
Effects of *Artemisia iwayomogi* extract supplementation on genes regulating adipogenesis in epididymal adipose tissue of mice fed with high-fat diet. (a) Representative example of semiquantitative RT-PCR revealing the expression levels of adipogenic genes in epididymal adipose tissue and their quantitative analysis. The data represent relative density normalized to GAPDH. Data represent the results of 3 independent experiments. Bars represent mean ± SEM. Means without a common letter differ, *P* < 0.05.

Examination of adipogenic gene expression in epididymal adipose tissue showed that mRNA levels of PPAR*γ*2 (−44%) and C/EBP*α* (−18%), regulators of adipogenic molecules, were significantly lower in AED-fed mice than in HFD-fed mice. The expressions of PPAR*γ*2 target genes, including cluster of differentiation 36 (CD36) (−29%), adipocyte fatty acid binding protein (aP2) (−35%), and fatty acid synthase (FAS) (−58%), were all significantly decreased in AED-fed mice than in HFD-fed mice (Figure 5). 

### 3.6. Expression of Genes Related to Inflammation

**Figure 6:**
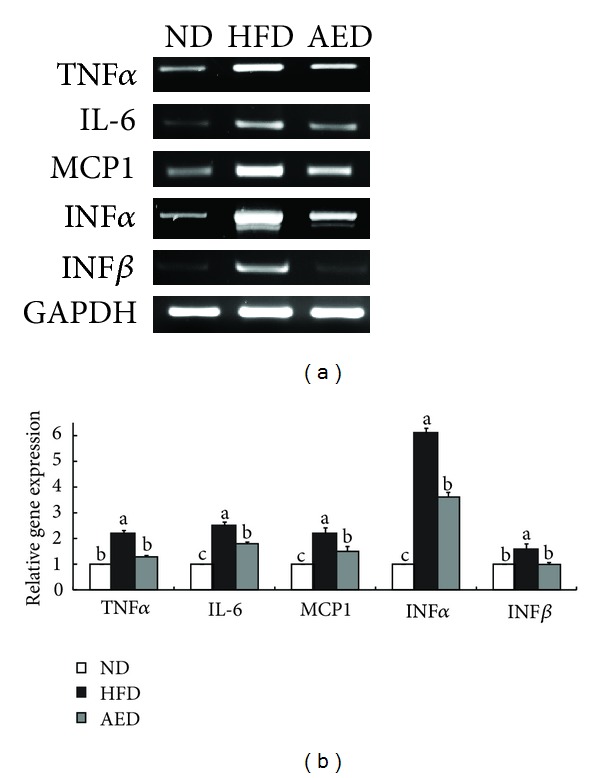
Effects of *Artemisia iwayomogi* extract supplementation on expression of proinflammatory cytokine genes in epididymal adipose tissue of mice fed with high-fat diet. (a) Representative example of semi-quantitative RT-PCR revealing the expression levels of proinflammatory cytokines in epididymal adipose tissue and their quantitative analysis. Data represent the results of 3 independent experiments. Bars represent mean ± SEM. Means without a common letter differ, *P* < 0.05.

Based on the active roles of proinflammatory cytokines in obesity-related inflammation, we examined the effect of AI extract on proinflammatory cytokine expressions in epididymal adipose tissue. Compared to HFD-fed mice, the epididymal adipose tissue in AED-fed mice contained significantly decreased mRNA levels of several proinflammatory cytokines, including TNF*α* (−41%), IL-6 (−28%), MCP1 (−32%), IFN*α* (−41%), and IFN*β* (−38%) (Figure 6). 

## 4. Discussion

This feeding study was designed to assess whether AI extract supplementation for 11 weeks could improve diet-induced obesity and obesity-related biomarkers in mice. Based on a preliminary study involving different AI extract dosages (0.5%, 1%, and 2%), we determined that 0.5% AI extract was the minimal effective dose for preventing weight gain in HFD-fed mice (data not shown). Hence, the 0.5% AI extract was considered for this study. In the present study, the AI extract significantly decreased not only body weight gain, but also visceral adiposity and adipocyte hypertrophy in HFD-fed mice. Since visceral adiposity is positively correlated to plasma leptin concentration, the circulating leptin level is an ideal indicator for assessing obesity in both experimental animals and humans [20, 21]. In this context, the lower plasma leptin level recorded in the AED-fed mice may be attributable to the prevention of visceral adipocyte hypertrophy.

As there was no significant difference in food consumption between HFD- and AED-fed mice, the beneficial effects of the AI extract on body weight gain and visceral fat accumulation evidently did not depend on decreased energy intake. Thus, we hypothesized that the AI extract reduced HFD-induced body weight gain and visceral fat accumulation by mediating the inhibition of adipogenesis. That the expression of PPAR*γ*2 and C/EBP*α* in WAT is upregulated in HFD-induced obese animals is well known [4, 22]. Increased PPAR*γ*2 and C/EBP*α* function cooperatively to transactivate adipocyte genes, including FAS, CD36, and aP2 [23–26]. FAS catalyzes the reactions for the synthesis of long-chain fatty acids [27], whereas CD36 and aP2 facilitate the uptake of long-chain fatty acids in adipocytes [23, 28], thereby increasing adipocyte size and fat accumulation. In the current study, the AI extract significantly reversed the HFD-induced upregulation of adipogenic transcription factors (PPAR*γ*2 and C/EBP*α*) and their target genes (FAS, CD36, and aP2) in the epididymal adipose tissue of mice. These decreased expressions of adipogenic genes by the AI extract may have contributed to the lower visceral adiposity and body weight gain. In addition, the AI extract significantly decreased plasma FFA levels in HFD-fed mice. As increased plasma concentrations of FFA form a vicious relationship with PPAR*γ*2 activation in diet-induced obese animals [29, 30], the decreased FFA level in the AED-fed mice might also be considered associated with PPAR*γ*2 inactivation.

Increased adipose tissue accumulation in obese individuals correlates with the overproduction of proinflammatory cytokines that play crucial roles in the development of obesity-induced inflammation [8, 11]. In obese animals, excess adipose tissue increases the expression and secretion of TNF*α*, a prototypical inflammatory cytokine [4, 9, 12]. In turn, increased TNF*α* activates adipocytes, thereby further enhancing the expression of various proinflammatory genes such as MCP1, IL-6, IFN*α*, and IFN*β* [31, 32]. IL-6 induces a hepatic acute-phase reaction with upregulated acute-phase proteins, including C-reactive protein and fibrinogen [33, 34], whereas MCP1 contributes to macrophage infiltration into adipose tissue, which leads to chronic inflammation [35]. IFN*α*/*β* directly stimulates IFN*γ* production, a regulator of innate immune response, in T cells [36]. Our results suggested that the decrease in visceral adiposity by the AI extract might have contributed, in part, to the decreased expression of proinflammatory cytokines (TNF*α*, MCP1, IL-6, IFN*α*, and IFN*β*) and reduced plasma levels of TNF*α* and MCP1. Consequently, the decreased gene expression and secretion of proinflammatory cytokines in the AED-fed mice may have contributed to the prevention of obesity-induced inflammation. 

Several studies have demonstrated that cytokines play crucial roles in the development of insulin resistance [35, 37–39]. In diet-induced obese animals, increased plasma levels of TNF*α*, IL-6, MCP1, and leptin have been shown to impair the ability of insulin to activate signal transduction and stimulate glucose uptake into skeletal muscle and adipose tissue [35, 37–41]. Thus, in the present study, improvement of insulin resistance by the AI extract might be associated with decreased gene expression and/or production of cytokines such as TNF*α*, IL-6, MCP1, and leptin.

Separation and determination of chemical constituents are generally recommended for standardization and quality control of herbal products and herb-related investigations [42]. Several studies have demonstrated that AI contains flavonoids such as genkwanin and jaceosidin [43], essential oils, including eugenol and 1,8-cineole [44], and coumarin compounds such as scopoletin and scopolin [43, 45]. In addition, scopoletin has been used as a standard compound for the verification and identification of AI [46, 47]. In the present study, we isolated not only scopoletin, but also scopolin and acetophenone glycoside from the AI extract (Figure 1 and Table 2). Of these, scopolin (1.21%) was the most abundant, followed by scopoletin (0.38%) and acetophenone glycoside (0.26%). In addition, the scopolin content of the AI extract was higher in our study than that observed by Kim et al. (0.49% w/w) and Ding et al. (0.2% w/w) [43, 45]. This difference may be due to the differing extraction and isolation methods as well as differences in plant material [43, 45]. Previous studies have shown that coumarin compounds from *Fraxinus rhynchophylla* [48]*, Angelica gigas* [49], and *Ionidium suffruticosum* [50] inhibit adipocyte differentiation in 3T3-L1 cells and/or reduce body weight gain and plasma lipid levels in mice fed a high-fat diet. Thus, the antiobesity activity of AI extract observed in the present study may be attributable to the presence of high amounts of coumarin compounds in the plant. Further studies are needed to determine the major active compounds in AI extract that are responsible for decreased visceral adiposity and other obesity-related biomarkers.

In summary, the present study showed that AI extract reduced visceral fat accumulation in HFD-fed mice and improved the risk factors related to the metabolic syndrome, such as inflammation and insulin resistance. The evidence obtained in this study suggests that the beneficial effects of AI extract may be due to, at least in part, downregulation of the genes related to adipogenesis and inflammation in the visceral adipose tissue of mice. Therefore, dietary supplementation with this extract, if validated in human studies, may provide an adjunctive therapy for the prevention and/or treatment of obesity and metabolic syndrome. 

## Figures and Tables

**Table 1:** Primer sequences and RT-PCR conditions.

Gene description	Primers	Sequences (5′ →3′)	*T*_*m*_ (°C)	Size (bp)
Peroxisome proliferator-activated receptor *γ*2 (PPAR*γ*2)	F	TTCGGAATCAGCTCTGTGGA	55	148
	R	CCATTGGGTCAGCTCTTGTG		
CCAAT/enhancer binding protein *α* (C/EBP*α*)	F	AAGGCCAAGAAGTCGGTGGA	55	189
	R	CCATAGTGGAAGCCTGATGC		
Adipocyte protein 2 (aP2)	F	ACATGAAAGTGGGAGTG	55	128
	R	AAGTACTCTCTGACCGGATG		
Cluster of differentiation 36 (CD36)	F	ATGACGTGGCAAAGAACAGC	55	160
	R	GAAGGCTCAAAGATGCCTCC		
Fatty acid synthase (FAS)	F	TTGCCCGAGTCAGAGAACC	55	171
	R	CGTCCACAATAGCTTCATAGC		
Monocyte chemoattractant protein 1 (MCP1)	F	CCAGCAAGATGATCCCAATG	55	450
	R	CTTCTTGGGGTCAGCACAGA		
Interferon *α* (IFN*α*)	F	ATGGCTAG(G/A)CTCTGTGCTTTCCT	60.2	500
	R	GGGCTCTCCAGA(T/C)TTCTGCTCTG		
Interferon *β* (IFN*β*)	F	TGGAGCAGCTGAATGGAAAG	55	122
	R	GAGCATCTCTTGGATGGCAA		
Tumor necrosis factor *α* (TNF*α*)	F	TGTCTCAGCCTCTTCTCATT	55	156
	R	AGATGATCTGAGTGTGAGGG		
Intereukin 6 (IL-6)	F	TTGCCTTCTTGGGACTGATG	55	162
	R	CCACGATTTCCCAGAGAACA		
Glyceraldehyde-3-phosphate dehydrogenase (GAPDH)	F	AGAACATCATCCCTGCATCC	60	321
	R	TCCACCACCCTGTTGCTGTA		

**Table 2:** Profile of compounds in the *Artemisia iwayomogi* extract.

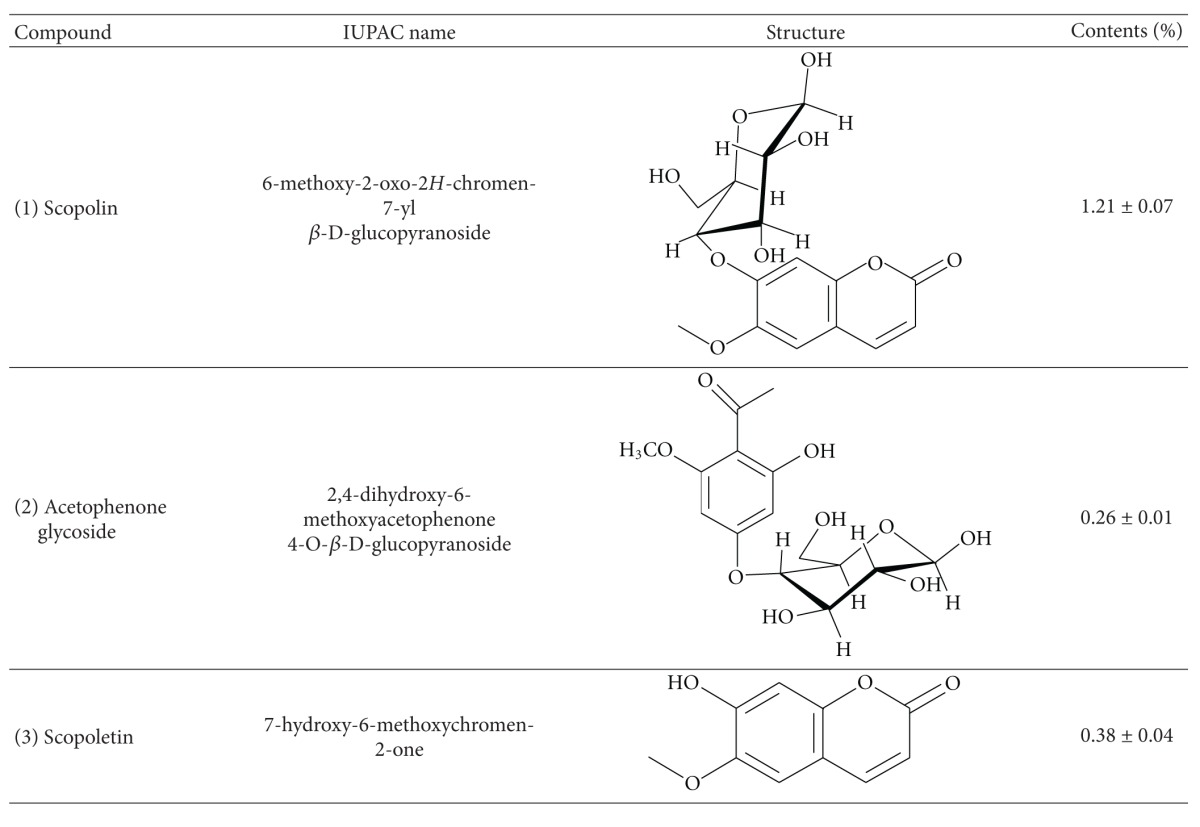

## Abbreviations

AI: *Artemisia iwayomogi*
AED: *Artemisia iwayomogi* extract-supplemented diet
aP2: Adipocyte protein 2
C/EBP*α*: CCAAT/enhancer binding protein *α*
CD36: Cluster of differentiation 36
FAS: Fatty acid synthase
FFA: Free fatty acids
GAPDH: Glyceraldehyde-3-phosphate dehydrogenase
HFD: High-fat diet
HOMA-IR: Homeostasis model assessment of insulin resistance
IFN*α*: Interferon *α*
IFN*β*: Interferon *β*
IFN*γ*: Interferon *γ*
IL-6: Interleukin-6
MCP1: Monocyte chemoattractant protein 1
ND: Normal diet
PPAR*γ*2: Peroxisome proliferator-activated receptor *γ*2
TG: Triglyceride
TNF*α*: Tumor necrosis factor *α*.
